# Alcohol and Hepatocellular Carcinoma: Adding Fuel to the Flame

**DOI:** 10.3390/cancers9100130

**Published:** 2017-09-25

**Authors:** Pierluigi Ramadori, Francisco Javier Cubero, Christian Liedtke, Christian Trautwein, Yulia A. Nevzorova

**Affiliations:** 1 Department of Internal Medicine III, University Hospital RWTH Aachen, Pauwelsstrasse 30, D-52074 Aachen, Germany; cliedtke@ukaachen.de (C.L.); ctrautwein@ukaachen.de (C.T.); 2 Department of Immunology, Complutense University School of Medicine, Madrid 28040, Spain; fcubero@ucm.es; 3 12 de Octubre Health Research Institute (imas12), Madrid 28041, Spain; 4 Department of Animal Physiology II, Faculty of Biology, Complutense University, Madrid 28040, Spain

**Keywords:** alcohol, hepatocellular carcinoma, acetaldehyde, oxidative stress, apoptosis

## Abstract

Primary tumors of the liver represent the fifth most common type of cancer in the world and the third leading cause of cancer-related death. Case-control studies from different countries report that chronic ethanol consumption is associated with an approximately 2-fold increased odds ratio for hepatocellular carcinoma (HCC). Despite the substantial epidemiologic data in humans demonstrating that chronic alcohol consumption is a major risk factor for HCC development, the pathways causing alcohol-induced liver cancer are poorly understood. In this overview, we summarize the epidemiological evidence for the association between alcohol and liver cancer, review the genetic, oncogenic, and epigenetic factors that drive HCC development synergistically with ethanol intake and discuss the essential molecular and metabolic pathways involved in alcohol-induced liver tumorigenesis.

## 1. Introduction: Alcohol Consumption and Its Impact on Human Health

Alcohol consumption has been shown to be causally associated with a large number of chronic diseases and deaths worldwide. Remarkably, it is estimated to be responsible for approximately 3.8% of all global deaths and almost 5% of the global burden of disease [1]. Europe represents the region with the highest overall alcohol consumption rate reaching on average twice the global average.

Alcohol abuse is causally associated with heart disease, stroke and vascular disease, as well as liver cirrhosis and cancer. In particular, about 10% of all cancer cases in men and 3% in women are directly attributable to alcohol consumption. In both genders, the alcohol-attributable fraction is relevant for the upper digestive tract (25–44%), colorectal carcinoma (4–17%), and liver cancer (18–33%) [2]. For example, case-control studies from different countries report that chronic ethanol consumption is associated with an approximately 2-fold increased odds ratio for the primary liver cancer- HCC. In this context, it is important to highlight that in the European Union, 60–80% of liver-related mortality is due to excessive drinking [3] and that alcohol-related chronic disease is the second most common indication for liver transplantation, accounting for approximately 40% of all primary liver transplants [4].

Recent studies have strengthened the evidence of carcinogenicity of alcohol by organs and by mechanisms of alcohol carcinogenesis even for low and moderate alcohol intakes. However, recent cohort studies indicate a linear dose-response relationship between average alcohol consumption and colorectal and liver cancers [5]. For example, the odds ratio increases to 5–7-fold with heavy ethanol intake exceeding 80 g/day for more than 10 years [6,7]. Notably, evidence has emerged that for some cancers the risk associated with alcohol actually decreases when consumption is reduced or interrupted [8].

In the context of alcoholic liver disease (ALD), clinical studies suggest that that the quantity and duration of alcohol consumption directly correlates with the stage of the disease [9,10]. Remarkably, in the Italian Dionysos study, which considered a wide cohort of northern Italian patients with non-viral chronic liver disease, highlighted that drinking without food, or drinking multiple types of alcoholic beverages, independent of the amount, is related to an increased prevalence of alcohol related liver disease [11]. This exemplary analysis focuses the attention not only on the overall quantity but also on the frequency patterns of alcohol consumption. How this aspect affects hepatic metabolism and the progression of liver disease is still unclear, although it is likely an essential consideration in order to develop new therapeutic approaches. Similarly, it is noteworthy that currently, in modern western societies, a great percentage of the young and adult population frequently consumes alcohol in combination with high caloric foods. It is, therefore, reasonable to consider that these individuals might be exposed to increased risk of detrimental effects, including metabolic syndrome and chronic alcohol consumption/dependence [12]. In fact, related clinical studies indicate that obesity and alcohol synergistically contribute to the progression of ALD and hepatocellular carcinoma development, although data in this regard it remains controversial [13,14,15,16,17].

Although epidemiological studies have validated a causal association of alcohol intake with several types of cancer, the underlying mechanisms through which alcohol triggers carcinogenesis are still not well understood. Moreover, how alcohol might drive the development and growth of certain cancers but not of others still represents an interesting area to investigate. Therefore, the main thrust of this review is to provide an update on both previous and current studies regarding alcohol and HCC molecular pathogenesis, to evaluate genetic and epigenetic risk factors, and to identify gaps in our current knowledge of alcohol-related tumorigenesis.

## 2. Alcohol Metabolism in the Liver

Alcohol is a small polar organic molecule that is able to diffuse through cell membranes and, via the bloodstream, can distribute into all tissues. However, whereas only a small part of ingested alcohol is absorbed and metabolized by the oxidative activity of several alcohol dehydrogenase (ADH) isoforms expressed in the stomach, the majority is metabolized in the liver [18,19].

**Figure 1:**
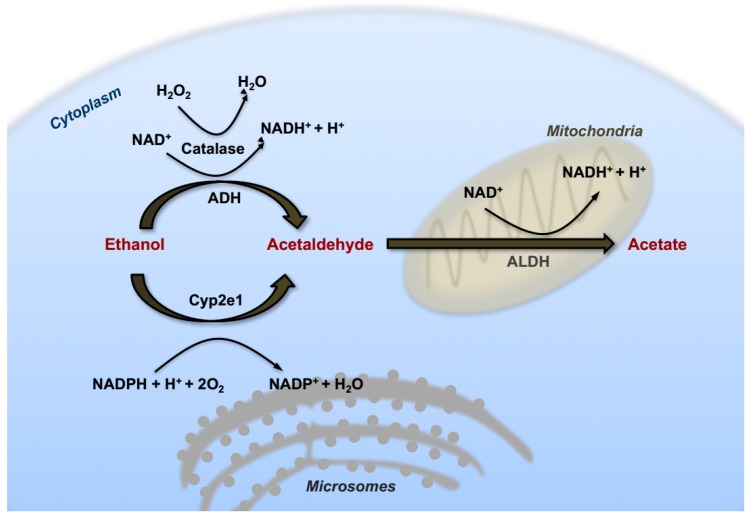
Hepatic alcohol metabolism. Schematic representation of the most important cellular pathways involved in ethanol metabolism localized in the different cellular districts. Alcohol is at first oxidized to acetaldehyde by alcohol dehydrogenase (ADH) in the cytosol or through the activation of the inducible microsomal enzyme CYP2E1. The heme-containing enzyme catalase can also participate to ethanol oxidation in the peroxisomes. Thereafter acetaldehyde is converted to acetate by the acetaldehyde dehydrogenase (ALDH) located in the mitochondria. Acetate can freely diffuse into the circulation.

In hepatocytes, the first catabolic step consists of an oxidative reaction catalyzed by the ADH in the cytosol converting ethanol into acetaldehyde that can diffuse into the intra-cellular space, and reach the bloodstream. This first reaction of alcohol catabolism (Figure 1) is responsible for the hydrogenation of the nicotinamide adenine dinucleotide (NAD) that generates a reduced form (NADH). Consequently, excessive increase of NADH/NAD^+^ ratio has been shown to contribute at least in part to the accumulation of triglycerides referred to as alcoholic steatosis. In fact, enhancing cytosolic reducing equivalents can influence several aspect of lipid metabolism via augmenting lipogenesis and inhibiting fatty acid oxidation (FAO) [19]. These redox changes, occurring during ethanol oxidation, affect also other aspects of hepatic metabolism, such as inhibition of gluconeogenesis, increased production of ketone bodies and exhaustion of cellular ATP reserves with increased hyperuricemia [20].

Another critical cellular compartment involved in ethanol catabolism is the microsomal ethanol oxidizing system (MEOS), containing an important member of the cytochrome P-450 family that was found to be inducible by chronic alcohol intake [21]. This component was identified as Cytochrome P450 family 2 subfamily E member 1 (CYP2E1). In fact, similarly to ADH, in response to high concentrations of ethanol CYP2E1 also actively participates in the process of ethanol oxidation. In contrast with ADH however, mRNA and protein levels of this cytochrome system are strongly upregulated in the liver of alcohol-drinking patients, and in experimental animals following chronic ethanol feeding [22,23]. CYP2E1 has a high redox potential and the catalytic reaction operated by this enzyme represents an important source of hydroxyl radicals, superoxide anion and hydroxyethyl radicals. In turn, boosting CYP2E1 activity results in a dramatic rise in intracellular oxidative stress and lipid peroxidation [24]. Experiments with transgenic and knockout mice further confirmed that CYP2E1 is required for genotoxicity via ethanol-mediated induction of direct oxidative stress to DNA in hepatocytes [25,26]. For these reasons, it has classically been considered a critical player in the contribution of excess alcohol intake and metabolism to liver carcinogenesis.

The enzyme catalase is an anti-oxidant enzyme typically acting as a peroxide (H_2_O_2_) scavenger that also takes part in ethanol catabolism by catalyzing the oxidation of alcohol to acetaldehyde in the peroxisomal compartment of hepatocytes (Figure 1) [27].

In a successive oxidative reaction (Figure 1), acetaldehyde is transformed into acetate by the enzyme acetaldehyde dehydrogenase (ALDH), mainly in mitochondria. Subsequently, acetate is converted spontaneously into water and carbon dioxide (CO_2_), and excreted. Lack of a typical mitochondrial isoform of the ALDH2 enzyme in 25–50% of the East-Asian population is responsible for the typical facial flush easily observable after intake of even small amounts of alcohol [28]. Interestingly, loss of functional ALDH2 results in attenuation of liver steatosis and lower serum transaminase levels, but paradoxically, aggravation of liver inflammation and fibrosis [29].

Chronic alcohol consumption leads to an over-saturation of these enzymatic systems, causing abnormal accumulation of acetaldehyde with consequent cytotoxic effects. Notably, pure ethanol does not directly induce cellular damage, but rather its hepatotoxic catabolic bi-products can trigger liver injury, inflammation and cell death. In this context, acetaldehyde toxicity can be attributed to its capacity to form DNA/protein adducts, giving rise to antibody production and enzyme inactivation [30]. More importantly, it exerts its mutagenic and carcinogenic potential though directly inducing DNA damage or by interfering with DNA synthesis and impairing activity of proteins involved in DNA repair [31]. A single molecule of acetaldehyde can react with DNA directly through the amino group of deoxyguanosine to generate a typical Schiff-Base adduct named *N^2^*-ethylidenedeoxyguanosine (*N*^2^-ethylidene-dGuo) [32]. Lack of a specific DNA repair pathway for this damage indicates a mutagenic potential for this kind of adduct. Another class of adducts commonly detected in the liver of alcoholic patients is propanodeoxyguanosine (PdG), resulting from the reaction of two molecules of acetaldehyde with DNA. PdG is suggested to exert mutagenic effects, although the actual cellular mechanisms are still unclear [33]. Moreover, it was shown that acetaldehyde can inhibit the activity and expression of the *O*^6^-methylguanine methyltransferase (MGMT), an enzyme responsible for DNA repair against alkylation [34]. Acetaldehyde can further increase cytotoxicity through enhancing generation of oxidative stress, which favors lipid peroxidation. These effects are mediated by depletion of mitochondrial glutathione (GSH), an important anti-oxidant system, possibly due to the formation of cysteine-adducts or inhibition of GSH up-take [35].

## 3. Pre-Carcinogenic Alterations of Hepatic Metabolism: From Steatosis to Oxidative Stress

The effects of alcohol on liver pathophysiology include a wide-spectrum of clinical features that are classically grouped under the general term of alcoholic liver disease (ALD). In fact, chronic alcohol consumption leads to changes of hepatic metabolism that, in association with other factors like sex, genetics or dietary habits progressively develop through simple steatosis, alcoholic hepatitis and fibrosis, to cirrhosis, with 1–2% of cirrhotic patients progressing to HCC, as end-stage liver disease. As outlined in the previous section, alcohol affects various aspects of hepatic metabolism through direct and indirect effects. At first, the reactions operated by the enzymatic systems ADH and ALDH lead to an increased intracellular NADH/NAD^+^ ratio, affecting important metabolic pathways including fatty acid oxidation and lipogenesis.

**Figure 2:**
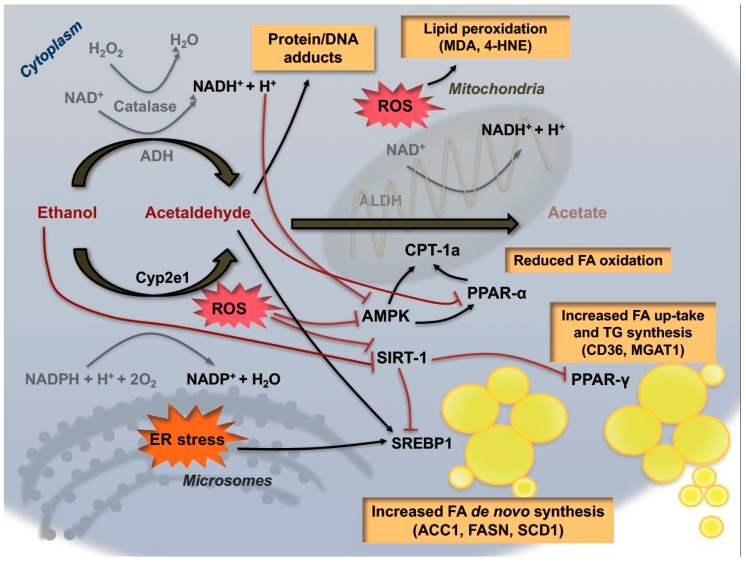
Metabolic effects of alcohol exposure on hepatocytes. Ethanol consumption directly and indirectly enhances lipid accumulation in hepatocytes via promoting the transcriptional activity of SREBP-1 and PPAR-γ. Acetaldehyde and ER stress also contribute to steatosis by inducing SREBP-1 activity and inhibiting PPAR-α expression. Moreover, alcohol metabolism increases ROS production via induction of CYP2E1 and impairment of mitochondrial functionality. Acetaldehyde and ROS exerts cyto-toxicity through generation of protein/DNA adducts and lipid peroxidation. ACC1: Acetyl-CoA-carboxylase-1; FASN: Fatty acid synthase; SCD1: Stearoyl-CoA desaturase-1; CPT1a: Carnitine Palmitoyltransferase 1a; CD36: Cluster of differentiation 36; MGAT1: Mannosyl(alpha-1,3)-glycoprotein beta-1,2-*N*-Acetylglucosaminyltransferase.

Although these changes in the redox balance might be sufficient to contribute to alcoholic liver steatosis, additional mechanisms have recently been proposed to increase hepatic triglyceride content (Figure 2). The pathways contributing hepatic steatosis and how this metabolic reprogramming might influence disease progression and progression to cancer are still poorly understood. In vitro studies have shown that ethanol reduces lipid catabolism via inhibition of β-oxidation in hepatocytes, and increased lipid biosynthesis and fatty acid up-take [36,37,38]. In this process, many factors participate in the metabolic changes occurring following alcohol intake. Acetaldehyde was shown to interfere directly with the transcriptional activity of two important transcription factors involved in lipid metabolism, notably PPAR (Peroxisome proliferator-activated receptors) family members and SREBP-1 (Sterol regulatory element binding protein 1). PPAR-α, a nuclear receptor known to be a major regulator of FAO, is negatively regulated by ethanol consumption, while pharmacological agonists of this nuclear receptor were shown to exert protective effects in the context of ALD [39,40]. Conversely, another member of the PPARs family, PPAR-γ, transcriptionally regulates genes responsible for fatty acid synthesis and uptake. Accordingly, several recent experimental studies shed light on the therapeutic potential of targeting of PPAR-γ in the context of alcoholic steatosis [41]. In particular, epigenetic modifications of this transcription factor, such as increased acetylation, have been proposed to be responsible for its activation during chronic alcohol consumption [42]. Interestingly, activity of Sirtuin-1 (SIRT-1), a NAD^+^-dependent deacetylase, is strongly inhibited by ethanol, and this has been shown to induce a reduction of PPAR-γ deacetylation, thereby enhancing its transcription. Similarly, the transcriptional activity of SREBP-1 is also increased during alcohol consumption through epigenetic modifications leading to up-regulation of genes involved in lipogenesis [43]. Chronic ethanol exposure can also reduce the activity of AMPK (5′ AMP-activated protein kinase), a critical regulator of cellular homeostasis, contributing to increase hepatic lipogenesis and decreased gluconeogenesis [44].

Lipid droplets’ turnover is a dynamic cellular process partly regulated by autophagy, a conserved mechanism enabling degradation and recycling of damaged cellular components such as proteins in order to promote efficient cell functionality. Ethanol consumption additionally influences autophagy in hepatocytes in different ways depending on the duration, and possibly amount, of alcohol administration [45]. Acute alcohol intake has been shown to enhance autophagy via FoxO3a activation [46] whereas chronic alcohol consumption appears to inhibit the autophagic process in a CYP2E1-dependent manner therefore contributing to lipid droplet accumulation in the liver [47]. Moreover, a particular form of autophagy occurring in the mitochondrial compartment, defined as mitophagy, recently emerged as a cytoprotective mechanisms against alcoholic mitochondrial damage-favoring mitochondrial turnover and improving β-oxidation, as demonstrated in Parkin-deficient mice [48].

In the context of inter-organ communication, alcohol was shown to decrease adipose tissue depots via increased CYP2E1 activation and oxidative stress. Cellular stress triggers a massive increase of lipolysis in adipocytes, possibly mediated by the hormone fibroblast growth factor-21 (FGF-21), with consequent release of fatty acid in the circulation, so contributing to hepatic steatosis [49]. Uptake of fatty acids therefore represents an important metabolic mechanism in alcohol-induced steatosis, as demonstrated in mice lacking the fatty acid transporter CD36. These mice actually display reduced triglyceride accumulation following ethanol feeding [50]. So far, the significance of hepatic fatty acid accumulation during alcohol consumption and how these metabolic changes impact on cell death and proliferation in the progression of the disease still remain elusive. Of note, genetic targeting of lipid droplet associated proteins like Perilipin-2 (Plin2) and Fat-specific protein (Fsp27) have been reported to successfully attenuate steatosis and related features of steatohepatitis in murine models of alcohol feeding [51,52]. Furthermore, recent interesting work by Bin Gao’s group [29] indicated that lack of functional ALDH2 in mice results not only in amelioration of steatosis, but also in an enhanced inflammatory response. This work suggests a possible dissociation of these two features in the progression of the disease.

Although a causal link with the hepatic increase of lipid accumulation is still to be elucidated, a key role for oxidative stress in the cytotoxic and mutagenic effects derived from alcohol consumption shows recent consolidation. As previously mentioned, induction of CYP2E1 activity and the exhaustion of mitochondrial GSH stores induced by accumulation of acetaldehyde are the most relevant and abundant sources of free radicals responsible for the increased oxidative stress triggered by chronic alcohol exposure. Ethanol can also react with hydroxyl radicals to generate 1-hydroxyethyl radicals (HER), a very reactive molecule that binds to protein-generating adducts which lead to organellar damage and mitochondrial dysfunction [53]. A vicious circle ensues, since reduced mitochondrial functionality represents in turn an important source of ROS production. Moreover, alcohol consumption can induce increased hepatic iron concentrations, as observed in in vitro experiments and in alcoholic patients [54], a phenomenon also contributing to ROS generation. Notably, chronic alcohol consumption in human and in experimental animals was shown to decrease tissue oxygen tension, with development of hypoxic areas in the liver contributing to ROS generation via activation of xanthine oxidase [55]. Together with altered metabolism of methionine, ethanol-dependent hypoxia might also impair the activity of the methionine adenosyltranferase leading to depletion of S-adenylmethionine (SAMe), a metabolic precursor of glutathione [56]. Accordingly, several studies have shown that administration of exogenous SAM exerts hepatoprotective effects, ameliorating steatosis and alcohol-related liver injury [57].

ROS generated via multiple sources can therefore, as a consequence of hepatic ethanol metabolism, react with several cellular components, and result in: lipid peroxidation, DNA mutation and enzyme inactivation-with consequent cellular damage and death. Aldehydes are bio-products of lipid peroxidation that, similarly to acetaldehyde, can form adducts with DNA, and are able to generate mutations of onco-suppressor genes, or oncogenes. In this regard, the aldehydes malondialdheyde (MDA) and 4-hydroxynonenal (4-HNE) were shown to exert mutagenic potential on the *p53* gene through reaction with deoxy-guanosine, deoxy-adenosine and deoxy-cytidine residues in DNA [58]. Furthermore, aldehydes can also react with protein-forming complexes which are able to trigger immune reactions, so generating epitope-specific antibodies, typically associated with ALD development [59]. In fact, patients in advanced stages of alcoholic disease display detectable circulatory levels of antibodies directed against protein adducts with either MDA or HNE [60]. It is, therefore, a reasonable approach that in recent years many efforts have been undertaken to demonstrate the beneficial effects of antioxidant therapies in this specific context. For example, pharmacologic or genetic targeting of CYP2E1 activity revealed an efficient therapeutic strategy limiting ROS production, steatosis and apoptosis in experimental models of alcohol consumption [61]. Moreover, selective inhibition of CYP2E1 was shown to prevent cancer development in mice treated with a combination of alcohol and the hepatocarcinogen diethylnitrosamine (DEN) [62]. Recent experimental work in mice shed light on the therapeutic potential of targeting the nicotinamide adenine dinucleotide phosphate oxidase (NADPH) oxidase complex (NOXs family) in the context of ALD [63]; although neither the particular isoform that may play a major contributory role nor the cell-specific localization in the liver have not been fully clarified [64]. Pharmacologic and genetic activation of the anti-oxidant regulator nuclear factor (erythroid-derived 2)-like 2 (Nrf2) has been reported to protect against alcoholic steatosis and alcohol-induced liver injury. Activation of Nrf2 via natural activator sulforaphane has been shown to increase the anti-oxidant levels of GSH and reduce liver steatosis in a model of acute alcohol injury [65]. Nonetheless, mice lacking Nrf2 are more susceptible to alcohol-induced liver damage-displaying a Srebp1-dependet increase in steatosis, and a dramatic depletion of glutathione associated with increased mortality [66]. In the context of carcinogenesis however, Nrf2 was shown to be overexpressed in many tumor entities including HCC [67]. Considering the intimate relation between alcohol consumption and HCC development, experimental evidence analyzing a possible role of Nrf2 as a catalyst within this partnership is still absent.

Finally, ethanol consumption has been associated with reduced hepatic levels of retinol (Vitamin A) [68], and it has been widely reported that alcoholic patients display decreased plasma retinol levels related to the severity of liver disease [69]. Alcohol appears to be a competitive inhibitor of vitamin A oxidation to retinoic acid, as the metabolism of these two compounds share enzymes critical in their catabolism, such as alcohol dehydrogenase and acetaldehyde dehydrogenase [70]. Moreover, alcohol-induced cytochrome P450, particularly CYP2E1, enhances catabolism of vitamin A and retinoic acid, whereas it alters retinoid homeostasis by increasing vitamin A mobilization from liver to extrahepatic tissues. Decreased hepatic retinol content has been proposed to play a contribution to the development of HCC through mechanisms affecting apoptosis and cell proliferation [71,72].

## 4. Genetics and Epigenetics of Alcohol-Related Liver Cancer

As mentioned above, overwhelming evidence indicates that the oxidative metabolism of ethanol is the principal driver of alcohol-induced cytotoxicity. Consequently, genetic mutations affecting enzymes involved in this metabolic process strongly influence the carcinogenic potential of alcohol (summarized in Table 1). Human hepatic ADH is a zinc metalloenzyme composed by 5 different isoforms (ADH1-5), generated by the different association of eight different subunits [18,73]. Single nucleotide polymorphisms (SNPs) have been identified and are prevalent in three loci, ADH1-ADH3, resulting in increased activity of the enzyme [74]. Although these polymorphisms better associate with gastric, esophageal, and colon cancers, it remains unknown if the frequency of these events correlate with the severity of ALD, and overall with the development of HCC [75]. Similarly, the enzyme ALDH can also form several isoforms, encoded in humans by 19 functional *ALDH* genes [76]. *ALDH2* is the mitochondrial isoform primarily responsible for acetaldehyde oxidation, mainly in liver and stomach. Polymorphisms causing reduced activity of the enzyme have been also identified in the *ALDH*2 gene, resulting in increased accumulation of acetaldehyde [77]. In a Japanese cohort the presence of this polymorphism, in combination with an ADH2 SNP, was shown to correlate with the incidence of HCC in cirrhotic patients consuming alcohol [78]. In general though, a higher association of the defective ALDH2*2 polymorphism with increased incidence of alcohol-related cancers (esophageal; head and neck cancer) has been demonstrated [79]. Thus, wider clinical cohorts are necessary to confirm the involvement of ALDH2*2 in HCC development.

Polymorphisms have also been individuated within the coding region of the *CYP2E*1 gene. However studies on the influence of this mutation on the development of ALD and HCC reveal conflicting data. A recent meta-analysis performed by Liu et al. [80] indicates that the presence of a PstI/RsaI polymorphism within the *CYP2E1* gene, markedly altering its transcriptional activity, might increase the risk of HCC in combination with alcohol consumption.

The epigenetic regulator protein methylenetetrahydrofolate reductase (MTHFR) transforms 5,10-methylenetetrahydrolate into 5-methyltetrahydrofolate, the most important donor of methyl group for methionine synthesis and processes of DNA methylation. Folate availability is therefore important for epigenetic regulation of gene expression and DNA synthesis. A SNP identified in the *MTHFR* gene, referred to as C677T, results in a dramatic reduction of enzymatic activity. Importantly, C677T was reported to significantly increase the risk of developing HCC in a cohort of patients with alcoholic cirrhosis [81].

PNPLA3 (Patatin-like phospholipase 3 domain containing 3) is predominantly expressed in adipose tissue and is a member of the patatin-like phospholipase family of proteins, which share homology with the lipase patatin. Mammalian patatin-like phospholipases (*PNPLAs*) are involved in a number of processes, such as maintenance of membrane integrity, lipid turnover and regulation of energy homeostasis [82]. Several of these enzymes are lipid hydrolases with substrate specificity for triacylglycerols, phospholipids, and retinol esters. A recent genome-wide association study performed in European individuals identified an important SNP in the PNPLA3 gene designated as rs738409. This polymorphism turned out to be an important risk factor for developing HCC from alcohol-related cirrhosis when it occurred concomitantly with the rs58542926 variant of the TM6SF2 gene, involved in hepatic lipoprotein metabolism [83]. More importantly, clinical evidence indicates a tight association between PNPLA3 rs738409 and the HCC incidence in alcohol-related cirrhotic patients [84].

Another example of a genetic polymorphism associated with increased HCC incidence in ALD has been identified in the neurocan (*NCAN*) gene. Here, the frequency of the rs2228603 allele was significantly increased in HCC patients with ALD etiology, but not in HCV-infected patients. Interestingly, it was also shown that this variant influenced the plasma low-density lipoprotein (LDL) and triglyceride (TG) levels [85]. These association studies clearly point to a central role for lipid metabolism in ALD and its progression to liver cancer.

**Figure 3:**
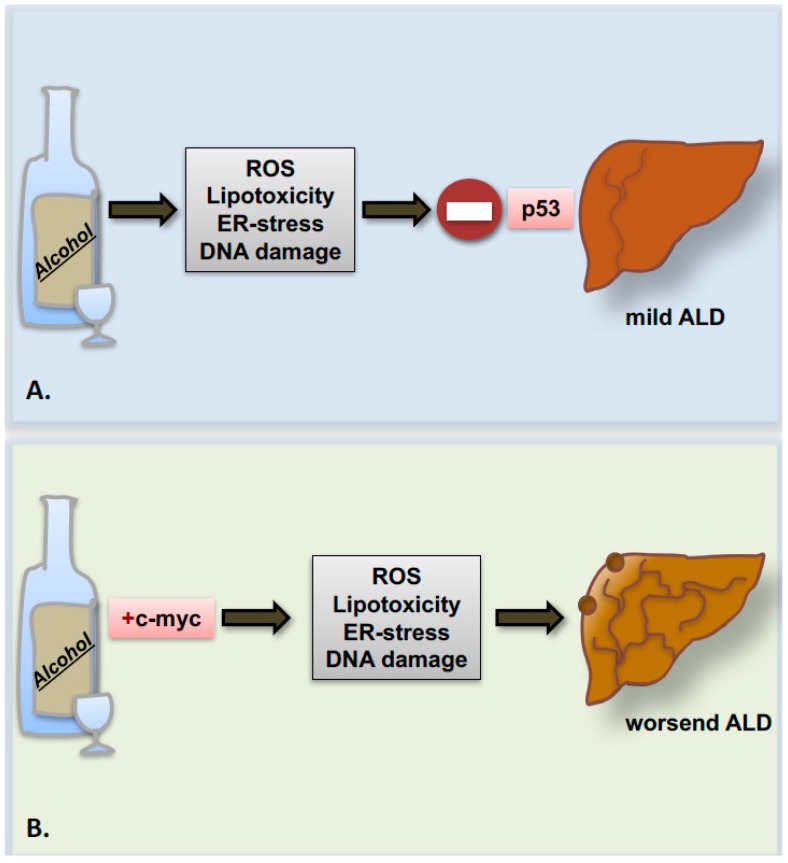
Enhanced expression of *c-myc* in liver promotes ALD progression. (**A**) Ethanol consumption leads to liver injury by generating ROS, lipid peroxidation, ER-stress and DNA-damage reactions: an effect buffered by “guardian” function of p53. (**B**) Alcohol intake and concomitant *c-myc* overexpression abrogate p53 activation and cause ALD progression to advanced precancerous stages.

Recently, a study from our group indicated that the proto-oncogene *c-myc* is strongly upregulated in patients with advanced stages of ALD. In parallel, in the experimental model of chronic ethanol feeding, overexpression of the proto-oncogene *c-myc* accelerates ALD progression to precancerous stages presumably though p53 inhibition (Figure 3) [86].

Chronic alcohol abuse affects also epigenetic mechanisms regulating gene expression. As we already illustrated, a decrease in methionine availability and therefore a reduced functionality of SAM synthase leads to a general reduction of transmethylation reactions altering the pattern of DNA methylation. Impaired methylation of gene promoters within several tumor suppressor genes has been observed in alcoholic patients with HCC [87]. For example, hypomethylation of *c-myc* and increased *c-myc* expression have also been observed in another experimental model of spontaneous alcohol-induced HCC [88].

Acetylation also represents an epigenetic mechanism that can be strongly altered by alcohol exposure. Hyperacetylation of a wide range of proteins has been reported in several experimental models of chronic alcohol consumption [89,90]. In particular, impaired acetylation of the Histone H3 complex might be responsible for liver injury following alcohol intake. As already mentioned, impaired acetylation due to alcohol-related SIRTs inhibition also contributes to the development of hepatic steatosis.

Dysregulation of the tumor suppressor PTEN (phosphatase and tensin homolog deleted on chromosome 10) has also been identified in chronic alcoholism and HCC. Redox-dependent modifications of the protein triggering a decrease in PTEN phosphatase activity has been shown to induce hepatic steatosis via Akt-activation in an experimental model of acute alcohol injury [91]. In contrast, chronic alcohol administration was reported to increase PTEN expression, thereby sensitizing hepatocytes to apoptosis through impairing Akt signaling [92]. Given that mice lacking PTEN display hepatomegaly, steatohepatitis, fibrosis and eventually resulting in HCC [93], further investigations are required to understand how genetic and environmental modulations of PTEN might contribute to the development of liver cancer in the context of ALD.

In the field of epigenetic regulation, increasing evidence indicates that alcohol influences the expression of proteins involved in different cellular functions via de-regulation of hepatic miRNAs (microRNAs) that can be released in the circulation upon hepatocyte death [94]. Particular attention has focused on the pro-inflammatory role played by the liver-specific miR-122 [95] and by miR-155 [96]. Furthermore, modulation of the expression of the oncogenic miRNA, miR-21, has also been associated with alcohol-induced liver injury and inflammation [97]. However, the rapidly growing area of microRNA research in the field of ALD is illustrated in more detail in a recent review [98].

## 5. Alcohol and the Cell Cycle Machinery: Hepatocyte Cell Death and Proliferation

The cytotoxic effects exerted by alcohol through the mechanisms described above induce cell death principally via activation of apoptosis. The apoptotic pathway is a caspase-dependent mechanism of cell death that triggers compensatory proliferation and thus increases the risk of mutational events. Apoptosis during alcohol exposure is a multi-step process involving mitochondrial dysfunction, increased oxidative stress, endoplasmic reticulum (ER) stress and impairment of autophagy [99,100]. ER stress is the result of the multifactorial consequences of alcohol metabolism on hepatocytes (described above), such as oxidative stress, acetaldehyde-dependent epigenetic modifications and alterations of SAM metabolism. In turn, activation of the unfolded protein response (UPR) triggers alteration of protein translation and folding, so compromising their functionality and degradation. Several markers of ER stress like the C/EBP homologous protein (CHOP), the glucose regulated protein 78 (GRP78), or the activating transcription factor-4 (ATF-4) were shown to be significantly over-expressed in livers of ALD patients and several experimental models of chronic alcohol consumption [101].

Overall, hepatocyte functionality is impaired by metabolism of excess ethanol on different cellular levels. Alcohol associated inflammatory cytokines (TNFα and FasL), stimulate the corresponding death receptors on hepatocytes, and subsequently trigger an apoptotic program executed through the extrinsic caspase cascade, ultimately leading to apoptotic cell death. The molecular mediators involved in activating the caspase-dependent execution process have been proposed to be related to the mitogen-activated protein kinase (MAPK/JNK) cascade [102]. Recent evidence indicate possible cross-talk between ROS, ER stress and the activation of the mitochondrial intrinsic apoptotic pathway [103]. Intrinsic pro-apoptotic members of the B-cell lymphoma 2 (Bcl-2)- family, oligomerise on the outer mitochondrial membrane and cause mitochondrial dysfunction. Following its release from the mitochondria, cytochrome c activates caspases responsible for the degradation of cellular substrates [104]. However, unpublished data from our own group suggest that alcohol represents a “mixed” type of apoptotic agent that employs both intrinsic and extrinsic signaling pathways [105]. In the context of oxidative stress production and apoptotic cell death, the consequent development of an inflammatory response via release of pro-inflammatory mediators represents a successive stage of the disease contributing to the amplification of hepatocyte cell death. It should be noted therefore that alcohol-induced cytotoxicity also results in necrosis and necroptosis. Whereas the first process develops mainly during the early and acute phase of damage [106], more attention has recently focused on an alternative mode of programmed cell death, referred to as necroptosis. Here, chronic ingestion of alcohol was shown to increase hepatic levels of receptor-interacting protein (RIP) 3, a central protein of the necroptotic complex, in mice and humans [107]. Accordingly, mice lacking RIP3 are protected from alcohol-induced cell death and steatosis [108]. However, more studies are needed to establish the overall influence of necroptosis in the progression of ALD and its relation with the apoptotic pathway.

The importance of understanding the role of apoptosis in the progression of ALD lies also with its capacity to trigger cell proliferation as a compensatory mechanism. In this regard, few data regarding the effects of alcohol on hepatocytes’ cell cycle and proliferation are available, and remain quite controversial. Classical in vitro studies performed by Clemens and co-workers [109] indicated that alcohol promotes cell cycle arrest of hepatocytes in G2/M phase and senescence via increased accumulation of the phosphorylated inactive form of the cyclin-dependent kinase, p-Cdk2, and of the cyclin dependent kinase inhibitor, p21. The augmented levels of these proteins result in a direct effect of acetaldehyde, rather than a consequence of oxidative stress. In mice and humans, similar observations regarding the increase of p21 protein levels have been confirmed also by other groups in association with the severity of ALD [110,111]. Accordingly, in our recent work on c-myc transgenic mice upon alcohol feeding, we also observed disruption in cell cycle regulation, leading to impaired compensatory proliferation, hepatocyte hypertrophy and consequently, liver enlargement [86].

In association with the above-described effects of oxidative stress on DNA, severe impairment of cell cycle might be responsible for the deregulation of hepatocyte proliferation driving the development of HCC. Of note, a previous study indicated a proliferative hepatocyte potential in response to alcohol injury driven by a pro-inflammatory environment in conjunction with increased hepatic RAS activity [112]. Similarly, Diehl’s group showed that under conditions of induced hepatocellular senescence, as it occurs during chronic alcohol consumption, the proliferation of the oval cell compartment might compensate the increased turnover of damaged hepatocytes [113].

Therefore, it is currently not understood precisely how alcohol could trigger HCC development (involving excessive proliferation of hepatocytes/oval cells), and simultaneously inhibit the hepatic cell cycle. It has been hypothesized that ethanol per se might not be a tumor inducer but rather a tumor promoter. However, experimental proof of this concept is still incomplete. Accordingly, identifying the genetic factors and oncogenes that drive HCC development synergistically with ethanol intake would allow the design of optimized therapeutic strategies.

## 6. The Gut-Liver Axis: Alcohol Effects on Primary Tumor Development and on the Pro-Metastatic Niche

**Figure 4:**
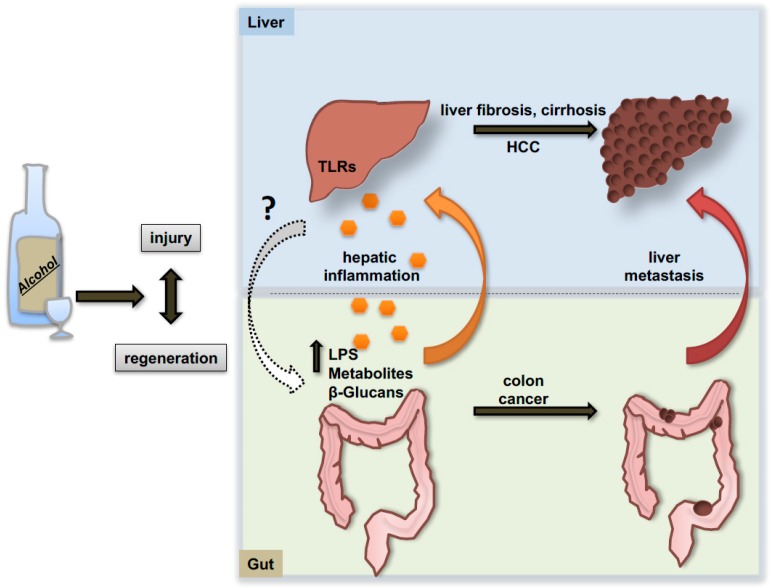
Gut-liver axis in the patho-physiology of alcoholic liver disease. Alcohol cyto-toxicity strongly affects the balance between hepatocyte death and proliferation through mechanisms not fully understood. Similarly, also the intestinal epithelial cells seem to be particularly susceptible to alcohol toxicity resulting in dysbiosis and alteration of intestinal barrier permeability. Bacterial and fungal products together with other metabolites released by the leaky gut contribute to exacerbate the inflammatory response in the liver by binding specific receptor on non-parenchymal hepatic cells, like toll-like receptors (TLRs) and other pattern recognition receptors (PRRs). In turn, injured and inflamed liver might influence intestinal epithelial cell survival and proliferation via systemic release of molecular mediators (left side of the slide). The alternation of alcohol-induce chronic damage and repair might favor the proper environment for the development of intestinal and liver cancers. Moreover, the inflammatory environment generating upon chronic alcohol consumption has been shown to enhance the migration of intestinal metastases towards the liver parenchyma (right side of the slide).

The progression of alcoholic liver disease is also influenced by the communication with other organs like the intestine. Consolidating evidence arose in recent years indicating that alcohol not only alters the quantitative and qualitative composition of the microbiome, but also induces alterations of the epithelial intestinal barrier with consequent release of bacteria and bacterial products that fuel the inflammatory response in the liver (Figure 4). It has been demonstrated that, upon alcohol exposure, lipopolysaccharide (LPS) and other bacterial products are released into the circulation and can bind to members of the toll-like receptors (TLRs) family on the cellular membrane of hepatic resident macrophages (Kupffer cells), thereby triggering the production of pro-inflammatory mediators in these cells [114]. In particular, mice lacking TLR4 displayed protection against steatohepatitis despite increased concentrations of plasma endotoxin following alcohol feeding [115]. Recent evidence in this regard shows that fecal microbiota transplantation and fecal microbiota manipulation via use of prebiotics might represent a valuable therapy against alcoholic liver injury and steatosis [116]. Similarly, dietary approaches and probiotic supplementations aimed at maintaining intestinal eubiosis revealed protective effects against ethanol-induced liver injury in mice and humans [117]. Moreover, interesting work from Schnabl’s laboratory sheds light on alterations of the mycobiome during alcohol consumption [118]. In fact, not only bacterial populations seem to be affected by ethanol intake but also the richness and the composition of intestinal fungi dramatically change under these conditions. Indeed, fungi can also be released in the circulation and behave as pathogen-associated molecular pattern molecules (DAMP/PAMP) binding specific receptors on Kupffer cells and promoting local IL-1β release.

Genetic depletion of the TLR4 receptor was subsequently shown to attenuate alcohol-associated steatosis and liver injury through MyD88-independent pathways [119]. In the context of cancer, TLR4 has been shown to interact with and activate the stem cell marker NANOG and induce liver tumorigenesis in mice fed high caloric diets. Accordingly, mice lacking TLR4 receptor developed much less tumors under the same dietary regimen [120]. A similar mechanism has been reported by Machida and co-workers indicating an interaction of TLR4/Nanog in tumor-initiating stem cell-like cells of the liver in a carcinogenetic combined model of alcohol consumption and HCV infection [121]. A connection between TLR4 and the microbiome in the context of HCC development has been elegantly demonstrated by Schwabe’s group in murine models of chronic liver injury [122]. Here, the authors demonstrated the efficiency of TLR4 depletion and the use of antibiotics or gut sterilization in limiting hepatic tumor promotion and formation.

The generation of a strong inflammatory environment derived from a direct effect of alcohol on non-parenchymal hepatic cells or indirectly fueled by the increased intestinal permeability, represents a condition that would amplify the process of liver injury and repair, and certainly favors the generation and proliferation of hepatic neoplastic foci (specific mediators of alcohol-induced inflammation are reviewed in detail elsewhere [123]). Additionally, a preclinical study of excess alcohol consumption indicates a 2.5-fold increase of hepatic metastases of colon cancer cells spreading from the spleen in ethanol-fed mice as compared to controls receiving water [124]. This enhanced aggressiveness is consistently reported to be associated with an enhanced inflammatory response and a systemic decrease of CD8^+^ and NKT^+^-cells [125].

In summary, recent pioneering work has identified gut-liver communication and the microbiome as important components involved in the development of ALD. However, intensive future investigations will be necessary to ascertain whether manipulating the intestinal microbiome might be a suitable approach to ameliorate or prevent ALD, and its progression to HCC.

## 7. Extra-Hepatic Effects of Alcohol

According to novel experimental and clinical findings, chronic alcohol intake appears to induce a series of systemic disturbances favoring optimal metabolic and immune conditions for the development of cancer. Beyond the effects on the gastro-intestinal tract that we described above, physiological homeostasis and functionality of other organs, such as white adipose tissue (WAT) and skeletal muscle, can be compromised influencing, in turn, the progression of liver disease. Supporting this, recent clinical and experimental findings suggest that chronic alcohol consumption reduces white adipose tissue mass via increased oxidative stress and inflammatory response [126]. This process induces a release of pro-inflammatory mediators (adipokines) that fuel the inflammatory response in liver. Moreover, mobilization of free fatty acids from the WAT due to excessive lipolysis is a potential source inducing hepatic steatosis [49]. In fact, the premise of a WAT-liver axis influencing metabolic and inflammatory alterations involved in ALD progression has recently emerged [127].

Moreover, acute alcohol intoxication or chronic alcohol consumption have been shown to induce atrophy of skeletal muscle, mostly related to a general impairment in protein synthesis, modulation of autophagy and reduction of the mTOR kinase activity [128]. Chronic alcohol exposure has been reported to induce muscle myopathy through altering the expression of genes involved in myogenic differentiation [129]. Autophagy seems also to be a factor in alcohol-induced sarcopenia. Indeed, increased expression of autophagy-related markers have been detected in skeletal muscle of alcoholic cirrhotic patients, as well as in the gastrocnemius of ethanol-fed mice, and murine myotubes exposed to ethanol treatment [130]. Interesting, inhibition of autophagy via knockdown of a central autophagic executer, Atg7, resulted in restoration of normal, physiological myotube size upon alcohol exposure. Furthermore, alcohol consumption results in reduced production of insulin-like growth factor (IGF-I) in muscle and liver with consequent alterations of muscular anabolic reactions and an imbalance of total protein turnover [131]. Thus, the injured or wasted muscle can produce inflammatory mediators (e.g., IL-6) that can in turn aggravate hepatic inflammation. It is noteworthy to consider that the cirrhotic patient frequently presents muscle loss related to malnutrition and metabolic deficiencies (e.g., Vitamin D) [132]. This could therefore indicate that beyond the direct effects of alcohol exposure on the skeletal muscle, the metabolic alterations of the chronic injured liver also indirectly impact muscular functionality.

## 8. Pre-Clinical Models of Alcohol Induced HCC

Given the multi-factorial complexity of alcoholic cytotoxicity and the interplay between both immune cells and hepatocytes and also between liver and other organs, there is an urgent need to re-create experimental conditions in vivo that reproduce clinical features of the alcoholic patient. Whereas in vitro studies analyzing the effects of alcohol on hepatocytes and hepatoma cell lines are quite exhaustive and mainly focused on aspects related to ethanol metabolism, in vivo pre-clinical models have recently been developed in order to evaluate the systemic influence of alcohol consumption in the context of hepatic carcinogenesis. Although representing a good reproducible model of chronic alcohol consumption, the typical experimental Lieber-Decarli liquid diet (using alcohol in combination with nutritionally adequate diets) did not, somewhat surprisingly, induce hepatic fibrogenesis and subsequent spontaneous hepatocarcinogenesis in mice. Thus, the Lieber-Decarli model per se is not suitable to mimic the pathogenesis normally observed in alcoholic patients with advanced stage of the disease. To the best of our knowledge, only two independent studies reported spontaneous development of liver neoplasia in alcohol-preferring rats (P-rats) after consumption of 10% alcohol in water for 18 months [133,134]. To this end, alcohol-induced injury protocols of chronic feeding in mice have been modified with combination of acute alcohol binge (NIAAA model) or high caloric feeding to increase liver injury and inflammation. Unfortunately, development of liver fibrosis and induction of carcinogenesis were not reported in these models.

Recently, multiple series of intraperitoneal diethylnitrosamine (DEN) injection followed by Lieber-Decarli diet feeding for up to 12 weeks has been adopted as a mouse model of HCC, promoted by alcohol consumption via alterations of the immune response [125]. Mice overexpressing the HCV Ns5a protein in hepatocytes develop HCC when fed long-term alcohol [120]. Also the combination of high caloric feeding with alcohol was reported to aggravate the effects of ethanol and induce liver fibrosis mainly through TLR4-dependent mechanisms, mimicking a clinical situation quite common in developed countries [135]. As this model recapitulates with good fidelity the human setting, a more consistent body of experimental evidences is required to understand how metabolic alterations driven by different dietary regimens influence the progression of the disease. It is quite well-demonstrated that fatty acids exert additive effects on alcohol-induced hepatic mitochondrial functionality, CYP2E1 activity and modulation of the immune response [136,137,138]. However, studies concerning the effects of diet composition at longer time-points on HCC development are still scarce and controversial [139,140,141].

It seems therefore necessary that further hepatic insults or a pro-inflammatory stimulus favor the conditions initiating cellular transformation in mice. Differences that are evident between experimental mouse models and the human condition, including genetic background, enzyme catabolic activity and environmental exposure to toxicant, should be considered in the interpretation and translation of data collected. Altogether, optimal animal models mimicking the different stages of the pathogenesis, from simple alcoholic steatohepatitis to liver fibrosis and HCC, are not yet available but are urgently required.

## 9. Conclusions

The studies reviewed here clearly indicate the complexity and multi-factorial etiology of ALD in relation to liver cancer. The effects of ethanol metabolism do not only impact the liver, but also involve a systemic and multi-organ crosstalk that inevitably contributes to a feedback loop that can aggravate the metabolic damage in hepatocytes. It is, therefore, crucial to understand all aspects of the disease in order to limit the tightly interconnected triggering events that irreversibly drive neoplastic alterations. Alcoholic liver damage represents a condition mainly related to psychosocial and cultural habits, and for this reason likely very difficult to eradicate. The combination of bad dietary habits, sedentary lifestyle and alcohol consumption represents a high-risk condition for developing chronic liver disease. Understanding how these factors interact at the metabolic level, in the progression of the disease, could indicate novel therapeutic/preventative approaches in the management of the alcoholic patient. Moreover, alcohol and high-caloric feeding have been shown to induce dysbiosis and alter intestinal permeability. In this regard, modulation of the intestinal microbiome is emerging as a promising therapy, and intensive studies are needed to establish safe and efficient intervention protocols. In the context of HCC, alcohol appears to be a promoter rather than initiator. Even so, the molecular pathways through which alcohol induces hepatocyte death are so far, still unclear.

Recent epidemiological data indicate that almost 5.8% of cancer deaths all over the world are attributable to alcohol consumption [2]. In times in which the enormous burden of infectious diseases, such as HCV, appears to be under increasing therapeutic control, more scientific efforts should focus on defining cellular pathways that could represent safe therapeutic targets in the limitation and management of chronic alcohol-induced liver damage.

## Figures and Tables

**Table 1:** Summary of the most frequent single nucleotide polymorphisms associated with ALD progression and HCC development.

Gene Name	Identified SNPs	Protein Functionality	ALD/HCC Association
**Alcohol dehydrogenase (ADH)**	ADH1B*2 (rs1229984) ADH1B*3 (rs2066702) ADH1C*1 ADH2*1 ADH2*2 (rs1229984) ADH3*2	Increased enzymatic activity	Associated with gastric cancers, but unknown association with ALD/HCC [74,75]
**Aldehyde dehydrogenase (ALDH)**	ALDH2*2 (rs671)	Reduced enzymatic activity	Correlation with HCC development in combination with ADH2*2 in a Japanese cohort [79]
**Cytochrome P450 Family 2 Subfamily E Member 1 (CYP2E1)**	PstI/RsaI (rs2031920/rs3813867)	Increased enzymatic activity	Association with HCC development in combination with alcohol consumption [80]
**Methylenetetrahydrofolate reductase (MTHFR)**	C677T (rs1801133)	Reduced enzymatic activity	Correlation with HCC in a population of alcohol-related cirrhotic patients [81]
**Patatin-like phospholipase 3 domain containing 3 (PNPLA3)**	I148M (rs738409)	Loss of enzymatic function	Important association with ALD progression and HCC development in alcohol-related cirrhotic patients [83], [84]
**Transmembrane 6 superfamily member 2 (TM6SF2)**	E167K (rs58542926)	Loss of expression and Function	Associated with HCC development in ALD setting in combination with I148M [83]
**Neurocan (NCAN)**	NCAN (rs2228603)	Altered functionality, unclear mechanisms	Association with HCC development in patients with ALD etiology [85]
